# 「児童・生徒作文コーパス」に対する節境界ラベル付与

**DOI:** 10.12688/f1000research.40669.1

**Published:** 2021-07-14

**Authors:** Mizuho Imada, Takumi Tagawa, Chang-Yun Moon, Akio Nasu

**Affiliations:** 1 Faculty of Humanities and Social Sciences, University of Tsukuba, Tsukuba, 305-8577, Japan

**Keywords:** 作文コーパス, 節境界ラベル, 書き言葉, 話し言葉, 言語発達, corpus of composition, clause boundary labels, written language, spoken language, language development

## Abstract

児童の作文能力の発達を評価するためには、単に係り受け距離などの計量的指標を評価するだけでなく、どのような種類の構造を使うかを考慮する必要がある。そこで「児童・生徒作文コーパス」に対してSVMによる節境界ラベル付与を行い、学齢の進行に伴う節の使用傾向の変化を調査した。文あたりの節ラベル頻度の分析では、連体節、補足節、引用節の増加と、並列節、条件節、理由節、時間節、間接疑問節の減少が見られた。係り受け距離の分析では、増加した節の多くは係り受け距離が小さく、減少した節の多くは係り受け距離が大きいこと、および学齢が上がるほど係り受け距離が小さい節の頻度が相対的に増大することを確認した。また、機能的に類似する節の間で、「て」から連用形へ、「たら」から「ば」へ、「けど」「けれど」から「が」へといった節選択の推移が見られた。これらの結果は、等位構造から従位構造への推移、話し言葉的な語彙から書き言葉的な語彙への推移という、児童の語彙・文法選択の変化を示唆する。

## Abstract (en)

To evaluate the development of children’s writing ability, it is necessary not only to examine quantitative indices such as the dependency distance, but also to inquiry the types of structures they use. We conducted clause boundary labeling using Support Vector Machine (SVM) on a corpus of Japanese students' compositions to investigate the change in the tendency of clause use with the progression of school age. The analysis of clause label frequency per sentence exhibited an increase in attributive clauses, nominal clauses, quotation clauses, and continuous clauses, and a decrease in parallel clauses, conditional clauses, reason clauses, time clauses, indirect interrogative clauses, and main clauses. The analysis of dependency distance demonstrated that most of the clauses that increased had short dependency distances, while most of the clauses that decreased had long dependency distances, and that the frequency of clauses with small dependency distances increased relatively with increasing school age. In addition, there was a shift in clause selection among functionally similar clauses, such as from “-te” to continuous forms, from “-tara” to “-ba”, and from “-kedo” and “-keredo” to “-ga”. These results suggest a change in the children’s lexical and grammatical choices, from coordinate to subordinate structures, and from spoken to written vocabulary.

## 1. 緒言

児童は学齢が上がり言語能力が発達するにつれて、より複雑な文を処理することができるようになると予測される。そこで児童の作文データを分析することによって文の複雑さを計量的に評価することを考えるが、そのためには文の複雑さを数値化する必要がある。比較的簡便に計量可能な指標としては文の長さ、係り受け距離、係り受けの深さなどがあり、基本的にはこれらの数値が大きいほど文の構造は複雑だと判断することができる。

しかし予測に反して、児童が学齢の上昇に従ってより複雑な文を書くようになると単純には言えないことを示すデータが存在する。低学年の児童は「〜して、〜して、〜して」のように並列節を多用して長い文を作ることがあり、このような文は「だらだら文」と呼ばれる。作文教育においては、長すぎる文の使用は好ましくないものとみなされており、短い文に分割するように指導されることが多い （ 松崎, 2016）。書き言葉の計量的な研究においては、小学生の作文における1文あたりの文字数、形態素数が学年によって大きくは増減しないこと （ 国立国語研究所, 1964: 386; 宮城・今田, 2015） ^1^が報告されている。これらの事実は、児童が成長につれてより複雑な文を書くようになるとは限らず、また複雑な文を書くことが直ちに作文能力の熟達を示すわけでもないことを示唆する。

児童の作文能力の統語的な発達を評価するためには、単に構造の複雑さを数値化するだけでなく、どのような構造が使われているかを考慮する必要がある。英語では、子供は作文の熟達に従って、口語的な接続詞を用いた等位構造の連鎖から、接続詞を多用せず従属節の使用、文や節の名詞化などにより情報が凝縮された節構造へと移行する （ Schleppegrell, 2004）。日本語の児童作文でも、逆接の「しかし」などを除けば接続詞は単調な増加傾向を示さず、一方で連体修飾成分が顕著な増加傾向を示す （ 宮城・今田, 2015）。また係り受け距離は、文節の種類によって頻度分布が異なる （ 張・尾関, 1996）。従って児童の統語能力の発達を評価するためには、構造の種類を無視して一律に複雑さを数値化するのではなく、発達に従ってどのような構造を用いるようになるかを重視しなければならない。

そこで本研究では、児童作文において使用される節の頻度分布を調査する。節は単文に相当する基本的な情報伝達単位であり、節を等位的に接続するか、埋め込みを多用するかといった構造の選択において、発達段階に応じた文法的方略の変化が観察されることが予測される。分析対象としては「児童・生徒作文コーパス」 （ 宮城・今田, 2015; 以下「作文コーパス」） を使用するが、節の位置と分類に関する情報 （節境界ラベル） は付与されていない。節境界ラベルを付与するシステムとしてはCBAP （ 丸山他, 2004）、Rainbow （ 加納他, 2014） があるが、一般に公開されていない。そのため本研究では「現代日本語書き言葉均衡コーパス」 （ BCCWJ, Maekawa et. al., 2014）の形態論情報と節境界ラベル （ BCCWJ-CBL, 丸山, 2013） を訓練データとして、汎用アノテーションツールYamCha （ Kudo and Matsumoto, 2003）で作文コーパスに節境界ラベルを付与した。

このデータを用いて児童作文における節の使用頻度を調査し、使用される節の種類が学年によってどのように変化するかを調べた。調査項目は、1文あたりの節ラベル頻度、節ラベル間の相対頻度、節の係り受け距離である。1文あたりの節ラベル頻度は、節の使用頻度を分析するための基本的な統計量である。節ラベル間の相対頻度は、理由節におけるカラ節とノデ節など、機能的に類似する節の相対的な選択嗜好の推移を分析するための統計量である。理由節全体の頻度が減少する場合、カラ節やノデ節の頻度も基本的に減少傾向が観察されるため、両者の選択嗜好を直感的に把握しやすくするために相対頻度を調べた。節の係り受け距離は、統語的特性の観点から節の使用傾向を分析するために調べた。平均的な係り受け距離は、並列節が多いほど大きく、連体節が多いほど小さくなると予想される。以下、データの作成と調査の方法を説明し、調査結果に対して検討を行う。なお、本研究で使用した作文データは、人名、組織名、地名など個人を識別できる情報があらかじめ被覆され、匿名化されている。また、公開したデータは節境界ラベルとテキスト中における位置情報のみを含み、個人を識別できる情報は含まれていない。従って出版の同意は必要なく、これにより科学的意味あいが歪められていない点を確認した。

## 2. 方法

### 2.1 SVMモデル

BCCWJ-CBLを教師データとして、YamChaを用いて節境界ラベルのSVMモデルを作成した。BCCWJ-CBLはBCCWJ本体とは独立したデータであり、節境界ラベルとラベル位置のみを含む。BCCWJには数値表現を解析しやすい漢数字表記に変換したNumTrans版 （NT） と原文のままの非NumTrans版 （OT） があるが、BCCWJ-CBLのラベル位置はNT版に基づく。一方、作文コーパスの形態論情報はNumTrans処理を行っていないOT形式のデータである。節境界付近に数値表現が現れることはあまり多くないと思われるが、OT形式でモデルを作成した方が作文コーパスにアノテーションをする際に精度がよくなると考えられるため、まずBCCWJ-CBLのラベル位置をNTからOTに変換する処理を行った。併せて、ラベル位置の斉一性のため、句読点や括弧などの記号の直後にラベルがあるときは、その直前の語彙的形態素の直後にラベルを移動した。

次に変換したBCCWJ-CBLとBCCWJの形態論情報 （OT版） を重ね合わせ、8割を訓練データ、2割をテストデータとしてYamCha形式のデータを作成した。YamChaで訓練データを学習させてSVMモデルを作成し、テストデータにアノテーションを行って精度を評価した （ 図 1）。使用した形態論情報は、出現形、語彙素読み、品詞大分類、品詞中分類、活用形である。学習はYamChaのデフォルト設定で行った。すなわち、前後2形態素を含む範囲の形態論情報と直前2形態素の推定済みラベルを素性として使用した。精度の評価は元データのラベルと推定したラベルの種類と位置が一致した場合を正解とし、元データのラベルのうち正解した率を再現率 （recall）、推定したラベルのうち正解した率を適合率 （precision）、両者の調和平均をF値として以下の式で計算した。

$$F=\frac{2∙\text{Recall}∙\text{Precision}}{\text{Recall}+\text{Precision}}$$

**図 1:**
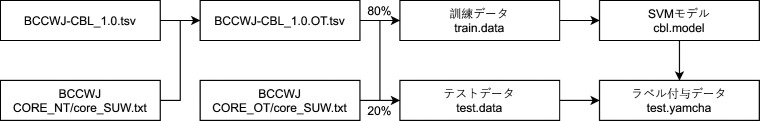
SVMモデルの作成と評価

### 2.2 節境界ラベル付与

前節と同様の方法で本番用のSVMモデルを作成し、作文コーパスに節境界ラベルを付与した。本研究で使用する作文コーパスは2014年から2016年までの3年間にかけて小学校全学年各2クラス、中学校全学年各4クラスの作文を悉皆的に収集・電子化した165万形態素規模のテキストコーパスである （ 表 1）。CaboCha/UniDicで形態論情報と係り受け情報を付与し、一部は人手により修正を施してある ^2^。まずBCCWJ-CBLの全データを使用して本番用のSVMモデルを作成した。次に作文コーパスのデータをYamCha形式に変換し、作成したSVMモデルでアノテーションを行った （ 図 2）。

**図 2:**
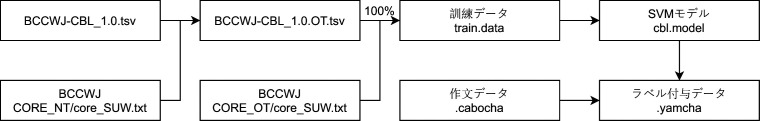
作文コーパスへの節境界ラベル付与

**表 1:** 「児童・生徒作文コーパス」の形態素数

survey_year	1	2	3	4	5	6	7	8	9	Total
2014	17941	25153	36376	53519	55189	52979	116199	114436	129582	601374
2015	17001	27473	34618	43232	47165	44381	110929	107291	112947	545037
2016	13388	21773	31243	35455	38318	41201	105605	116510	96662	500155
Total	48330	74399	102237	132206	140672	138561	332733	338237	339191	1646566

「児童・生徒作文コーパス」のデータ規模を形態素数で示す。学年は、1が小学校1年生、9が中学校3年生である。

BCCWJ-CBLのラベルは198種に及ぶが、この中には頻度が低く計量的な分析に適さないものも含まれる。そのため、付与したラベルをグループ化するためにcbl1 （大分類）、cbl2 （小分類）、cbl3 （接頭辞）、cbl4 （接尾辞） の4つの属性に分割したラベルを追加した。接頭辞は「文末」、接尾辞は「助詞」「補足語」を含む （ 表 2）。以下、分かりやすさのためにBCCWJ-CBLのラベルを鉤括弧、本研究で追加したラベルを二重鉤括弧で示す。

**表 2:** 節境界ラベルのグループ化

cbl (original)	cbl1	cbl2	cbl3	cbl4
文末_その他	主節	その他	文末	
時間節トキ	時間節	トキ		
タメニ節	連用節	タメニ		
連用節	連用節	動詞型		
時間節トキノ	連体節	トキノ		
ナド節-補足語	連用節	ナド		補足語
文末_間接疑問節-助詞	間接疑問節		文末	助詞

「文末」「文末_その他」はcbl1を「主節」とした。「時間節トキ」の形式のものは「時間節」をcbl1、「トキ」をcbl2とした。「タメニ節」の形式のものは統語的性質に応じてcbl1を「連用節」または「連体節」とした。「連用節」「連用中止節」「イ形容詞連用節」はそれぞれcbl2を「動詞型」「ナ形容詞型」「イ形容詞型」とした。引用節や時間節で連体的なものはcbl1を「連体節」とした。「文末_」「-助詞」「-補足語」などが付属するものは、それぞれcbl3、cbl4を「文末」「助詞」「補足語」とした。

### 2.3 文あたり節ラベル数 （clauses per sentence)

節境界ラベルを付与した作文コーパスを用いて、学年ごとの節の頻度を調べた。ラベルの頻度は作文の長さに比例して増加するため、頻度の調整が必要である。調整方法として、形態素数あたりの頻度と文数あたりの頻度が考えられるが、ここでは文数あたりの頻度で調整した。どちらの方法も文が長くなるほど「文末」以外のラベルの頻度が相対的に大きくなるが、後者の方法では「文末」ラベルがほぼ一定の値 （1文あたり1つ） を取るので、直感的に理解しやすいためである。1文あたりの節ラベル数をcps （clauses per sentence）とし、文書dにおける節境界ラベルcのcpsを以下のように定義する。 $s_{d}$は文書dにおける文数、 $n_{d,c}\;$は文書dにおける節境界ラベルcの頻度である。

$${\mathrm{cp}s}_{d,c}=\frac{n_{d,c}}{s_{d}}$$

この値を全ての文書d、節境界ラベルcについて計算し、学年によるcpsの変化を調べた。cpsは片側に裾の長い分布を取るため、実際の分析にはlog （cps）を使用した。まずcbl1レベルにおけるlog （cps）の分布を箱髭図で可視化し、次に以下のモデルで単回帰分析して各ラベルのlog （cps）の学年による傾きを求めた。

$$\log\left(\mathrm{cps}\right)〜1+\text{school}\_\text{year}$$

学年による傾きが正の値を取れば学年に従って頻度が増加し、負の値を取れば減少するものと解釈することができる。

### 2.4 節境界ラベルの相対頻度

単回帰分析の結果cpsの傾きが小さかったいくつかのcbl1について、その下位のcbl2から比較的頻度が高く、かつ機能的に類似するものをいくつか選択し、相対頻度を調べた。節境界ラベルcが複数の節境界ラベルC={c _1_, c _2_, …}に占める相対頻度 $r_{c}$は以下のように計算した。 $f_{c}$はcの頻度である。

$$r_{c}=\frac{f_{c}}{\sum_{x\in C}f_{x}}$$

rの値が大きいほど、節境界ラベルの集合Cの中で相対的に使用頻度が高いと解釈することができる。比較のために、まず各cbl2におけるlog （cps）をcbl1と同様のモデルで単回帰分析し、次に学年別の rを積み上げ棒グラフで可視化した。

### 2.5 節の係り受け距離

文節係り受け構造において、係り元の文節から係り先の文節までの文節数を係り受け距離 （dependency distance）とする。作文コーパスに付与されている係り受け情報は全て左から右への係り受けなので、文節sから文節tまでの係り受け距離 $dd_{s}$は両者の文節番号の差 $\mathrm{id}\left(t\right)-\mathrm{id}\left(s\right)$で計算することができる。文末文節は係り先を持たないので dd=0とする。

まず、節の種類によって係り先までの距離が異なることを確認するために、節境界ラベルが付与されている文節の係り受け距離を調べ、cbl1ごとに平均を求めた。次に、これらの文節の係り受け距離の分布が学年によってどう変化するかを調べるために単回帰分析を行った。係り受け距離の頻度分布はZipfの法則に従う （ Maruyama and Ogino, 1993; 金, 1996; 張・尾関, 1996） とされているので、距離 ddと頻度 $n_{\mathrm{dd}}$は $\log\left(n_{\mathrm{dd}}\right)=a+b\log\left(\mathrm{dd}\right)$に従うと考えられる。そのため、以下のモデルで学年ごとに単回帰分析を行った。 dd=0の文節は対数が取れないので除外した。

$$\log\left(n_{\mathrm{dd}}\right)〜\log\left(\mathrm{dd}\right)$$

bは負の値を取るが、この値が小さい （絶対値が大きい） ほど ddが小さい文節と大きい文節の頻度差が大きい、すなわち係り受け距離の短い文節が多いと解釈することができる。

## 3. 結果

### 3.1 SVMモデル

BCCWJ-CBLの8割を教師データ、2割をテストデータとしてアノテーション精度を評価した結果、適合率97.34%、再現率96.21%、F値96.77%だった。CBAP （ 丸山他, 2004） の解析精度はF値で97.32%〜98.87%とされており、既存の節境界ラベル付与プログラムと比べても大きく遜色ない精度が得られた。日本語の節境界は局所的な形態素列によってかなりよい精度で検出可能 （ 丸山他, 2004） であるため、SVMでも高い精度で検出できたものと考えられる。

### 3.2 節境界ラベル付与

BCCWJ-CBLの全データを教師データとしてSVMモデルを作成し、作文コーパスに節境界ラベルを付与した。付与したラベルの総数は203,152ラベル、使用したラベルはBCCWJ-CBLの198種中155種だった （ 表 3）。

**表 3:** 作文コーパスにおける節境界ラベルの頻度

cbl1	cbl2	total
主節	_ （70700）, その他 （5204）	75904
連用節	テ （17794）, 動詞型 （9966）, ヨウニ （3863）, テモ （1716）, ナ形容詞型 （1288）, タメニ （859）, ズニ （645）, ノニ （616）, ナガラ （581）, テカラ （548）, その他 （327）, ニハ （313）, タメニハ （281）, タメ （279）, ヨウ （258）, シニ （234）, ダケ （232）, テハ （232）, ナド （174）, イ形容詞型 （153）, ッテ （153）, マデ （153）, ホド （146）, ヨリ （131）, トカ （119）, ママ （73）, ナガラモ （58）, ツツ （37）, マデニ （37）, マデニハ （32）, マデハ （16）, ダケニ （8）, ニモ （8）, ドコロカ （7）, ホドニ （6）, ホカ （4）, マデモ （1）	41348
連体節	_ （20673）, トイウ （2061）, ヨウナ （736）, トキノ （209）, タメノ （138）, テノ （69）, ナドノ （48）, ホドノ （48）, ダケノ （37）, マデノ （36）, アトノ （26）, テカラノ （22）, カギリノ （18）, マエノ （7）, トノ （5）, バアイノ （1）	24134
引用節	_ （15049）, ッテ （67）, ナンテ （57）	15173
補足節	_ （12190）	12190
並列節	ガ （3847）, タリ （3778）, ケド （1379）, シ （1355）, ケレド （695）, ケレドモ （58）, デハ （2）	11114
条件節	ト （5099）, タラ （2621）, バ （2015）, ナラ （167）, モノノ （85）, カギリ （39）, ケッカ （38）, ナラバ （31）, バアイ （20）	10115
理由節	ノデ （4298）, カラ （1455）	5753
時間節	トキ （1655）, トキニ （609）, その他 （534）, トキニハ （118）, アト （116）, マエニ （105）, アトニ （43）, マエ （40）, イマ （15）, アトデ （3）, イライ （2）	3240
間接疑問節	_ （2419）, カドウカ （34）	2453
未定義	_ （1727）	1727
譲歩節	ニセヨ （1）	1

作文コーパスに自動付与した節境界ラベルの頻度を大分類 （cbl1） 、小分類 （cbl2） で集計した。cbl2の_はラベルなし、括弧内の数値は頻度である。

### 3.3 文あたり節ラベル数 （clauses per sentence)

大分類ごとのlog （cps）の分布を 図 3に、その単回帰分析の結果を 表 4に示す。譲歩節は1例しかないため、単回帰分析から除外した。単回帰分析に基づくlog （cps）の傾きを見ると『連用節』『補足節』『引用節』『連体節』は0.01〜0.07の傾きで増加しており、『条件節』『理由節』『並列節』『時間節』『未定義』『間接疑問節』『主節』は−0.09〜−0.01の傾きで減少している。『連用節』は5%水準で有意、それ以外は0.1%水準で有意だった。

**図 3:**
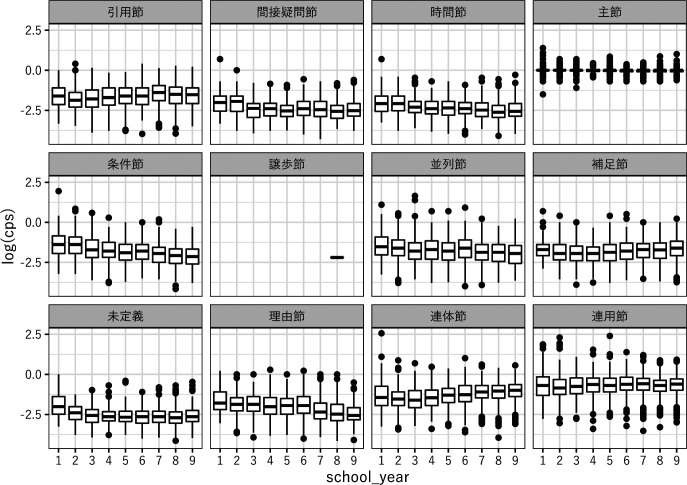
cbl1 におけるlog (cps) の分布

**表 4:** cbl1 におけるlog (cps) の単回帰分析

	引用節	間接疑問節	時間節	主節	条件節	並列節	補足節	未定義	理由節	連体節	連用節
(Intercept)	−1.75 ^***^	−2.25 ^***^	−2.09 ^***^	0.01	−1.34 ^***^	−1.53 ^***^	−1.95 ^***^	−2.36 ^***^	−1.58 ^***^	−1.66 ^***^	−0.74 ^***^
	(0.03)	(0.06)	(0.04)	(0.00)	(0.03)	(0.03)	(0.03)	(0.05)	(0.03)	(0.03)	(0.02)
school_year	0.02 ^***^	−0.03 ^***^	−0.05 ^***^	−0.01 ^***^	−0.09 ^***^	−0.05 ^***^	0.02 ^***^	−0.03 ^***^	−0.09 ^***^	0.07 ^***^	0.01 ^*^
	(0.00)	(0.01)	(0.01)	(0.00)	(0.00)	(0.01)	(0.00)	(0.01)	(0.01)	(0.00)	(0.00)
R ^2^	0.01	0.01	0.04	0.01	0.10	0.02	0.01	0.02	0.09	0.06	0.00
Adj. R ^2^	0.00	0.01	0.03	0.01	0.10	0.02	0.01	0.02	0.09	0.06	0.00
Num. obs.	4262	1467	2091	5324	4041	4013	4177	1314	2986	4740	5151

^***^p < 0.001; ^**^p < 0.01; ^*^p < 0.05.

### 3.4 節境界ラベルの相対頻度

cbl1の単回帰分析で傾きが小さかった4種類のcbl1に属する合計10種類のcbl2を選んで相対頻度を調べた。対象としたcbl1は『連用節』『理由節』『条件節』『並列節』4種類で、単回帰分析の傾きは−0.09〜0.01と比較的小さい。これらのcbl1に属するcbl2のうち、連用節の『動詞型』『テ』、理由節の『カラ』『ノデ』、条件節の『タラ』『ト』『バ』、並列節の『ガ』『ケド』『ケレド』を分析対象とした。これらは各cbl1で比較的頻度の高いcbl2であり、連用節の67%、理由節の100%、条件節の96%、並列節の53%を占める。並列節は『タリ』『シ』も頻度が高いが、逆接の『ガ』『ケド』『ケレド』とは機能が大きく異なるので除外した。

これらのcbl2をcbl1と同様に単回帰分析したところ、『動詞型』が有意な正の傾きを示し、『ガ』が有意な傾きを示さないことを除いて、他の全てのcbl2の傾きが有意に負の値を示した （ 表 5）。一方、これらのcbl2について、同じcbl1に属するものに分けて学年ごとに相対頻度 rを調べたところ、『動詞型』『ガ』『バ』は学年が上がるほど rが増加しており、『ノデ』『ケレド』も小学校高学年頃まで上昇が見られた （ 図 4）。

**図 4:**
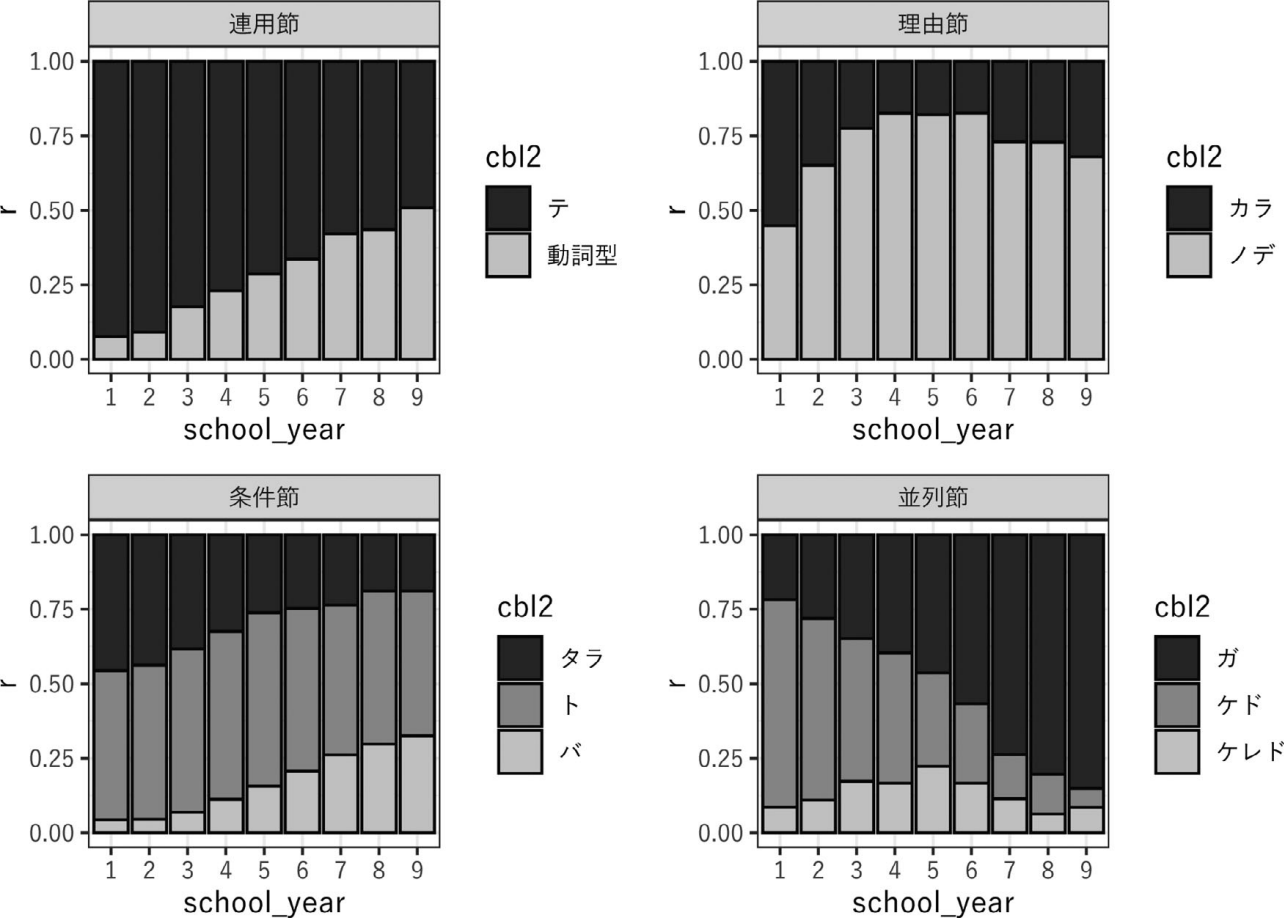
cbl2の相対頻度

**表 5:** cbl2におけるlog (cps) の単回帰分析

	条件節			並列節			理由節		連用節	
	タラ	ト	バ	ガ	ケド	ケレド	カラ	ノデ	テ	動詞型
(Intercept)	−1.64 ^***^	−1.60 ^***^	−2.38 ^***^	−2.36 ^***^	−1.81 ^***^	−2.12 ^***^	−1.83 ^***^	−1.71 ^***^	−0.89 ^***^	−2.19 ^***^
	(0.04)	(0.03)	(0.06)	(0.05)	(0.05)	(0.08)	(0.05)	(0.04)	(0.03)	(0.04)
school_year	−0.13 ^***^	−0.10 ^***^	−0.02 ^**^	0.00	−0.10 ^***^	−0.06 ^***^	−0.11 ^***^	−0.08 ^***^	−0.10 ^***^	0.04 ^***^
	(0.01)	(0.00)	(0.01)	(0.01)	(0.01)	(0.01)	(0.01)	(0.01)	(0.00)	(0.01)
R ^2^	0.20	0.13	0.01	0.00	0.11	0.05	0.15	0.06	0.10	0.02
Adj. R ^2^	0.20	0.12	0.01	−0.00	0.11	0.05	0.15	0.06	0.10	0.02
Num. obs.	1714	2794	1402	2140	963	532	1067	2337	4746	3468

^***^p < 0.001; ^**^p < 0.01; ^*^p < 0.05.

### 3.5 節の係り受け距離

cbl1ごとに係り受け距離の平均を計算したところ、『並列節』が4.38で最も大きく、『主節』が0.11で最も小さかった （ 表 6）。また $\log\left(n_{\mathrm{dd}}\right)$を学年ごとに単回帰分析した結果、 $\log\left(\mathrm{dd}\right)$による傾きは全学年を通じて負の値を取り、かつ学年が上がるほど小さい値をとる傾向があることがわかった （ 表 7）。ただし、小学2年、小学6年、中学3年では直前の学年より傾きが大きい値を取った。

**表 6:** cbl1の平均係り受け距離

cbl1	dd (mean)
並列節	4.73
理由節	4.03
条件節	3.88
時間節	3.42
譲歩節	3.00
連用節	2.93
未定義	2.28
補足節	2.10
間接疑問節	1.55
連体節	1.14
引用節	1.13
主節	0.05

**表 7:** 各学年におけるlog （n _dd_） の単回帰分析

	1	2	3	4	5	6	7	8	9
(Intercept)	8.09 ^***^	7.85 ^***^	9.41 ^***^	9.93 ^***^	10.08 ^***^	9.25 ^***^	11.40 ^***^	11.50 ^***^	11.40 ^***^
	(0.34)	(0.38)	(0.27)	(0.32)	(0.34)	(0.41)	(0.40)	(0.44)	(0.44)
log (dd)	−2.38 ^***^	−2.09 ^***^	−2.56 ^***^	−2.66 ^***^	−2.75 ^***^	−2.40 ^***^	−3.08 ^***^	−3.14 ^***^	−3.07 ^***^
	(0.13)	(0.12)	(0.09)	(0.11)	(0.12)	(0.13)	(0.15)	(0.16)	(0.17)
R ^2^	0.93	0.87	0.95	0.94	0.94	0.89	0.93	0.93	0.93
Adj. R ^2^	0.92	0.87	0.95	0.94	0.94	0.89	0.93	0.93	0.92
Num. obs.	30	48	38	36	34	42	34	31	29

p < 0.001; p < 0.01; p < 0.05.

## 4. 考察

### 4.1 等位構造から従位構造へ

cbl1におけるlog （cps）の単回帰分析 （ 表 4） から、学年の上昇に伴って『連体節』『補足節』『引用節』『連用節』が増加すること、『条件節』『理由節』『並列節』『時間節』『未定義』『間接疑問節』『主節』が減少することが分かる。『連用節』は5%水準で有意、それ以外は0.1%水準で有意だが、箱髭図を見ると必ずしも一様に増加・減少しているとは言えず、ラベルによっては増加する時期、減少する時期、ほとんど変化しない時期があるようである。

『連体節』『補足節』『間接疑問節』は名詞句を形成する節であり、『引用節』も名詞句を作るわけではないが補足節の一種とされる （ 現代日本語文法研究会, 2008）。これらの節は述語の項として機能し、典型的な従位構造である埋め込み文を形成する。このうち『連体節』『補足節』『引用節』は学年の上昇に従ってcpsが増大しており、学年が上がるほど従位構造を多用する傾向があることを示している。連体節は学齢に従って増加することが報告されており （ 小熊他, 1998）、本調査でも同様の傾向が確認できる。ただし箱髭図を見ると『連体節』『補足節』とも小 1 から小 3 にかけて若干減少し、その後増加している。また『引用節』も小 1 から小 2 にかけて減少した後増加し、さらに中 1 以降は再び減少している。このような複雑な変化は、単に学齢が上がるに従ってこれらの名詞的な節を多用するようになるという以外の何らかの文法的方略の変化が特定の時期に生じていることを示唆する。『間接疑問節』のみcpsが減少する傾向が見られるが、この原因についてはさらに研究する必要がある。

『並列節』は等位構造を作る節である。『条件節』『理由節』『時間節』は副詞節の一種とされ （ 現代日本語文法研究会, 2008）、意味機能的には従位的な性格を持つ節だが、名詞節や補足節が述語の項として機能するのと異なり、節と節が統語的には並列に接続するという点で等位構造に近い性質を持つ。これらの節は学年の上昇に従ってcpsが減少しており、学年が上がるほど等位構造の使用が減少することを示している。特に条件節と理由節は2 つの事柄の因果関係を表現する節であり、こうした関係を表現する能力は学齢に従って発達すると予測されるが、本調査の結果からは減少する傾向が見られる。この理由として、文を 2 つに分割して接続表現や指示表現で結束性を表現したり、名詞句や副詞句など別の手段で因果関係を表現する方略の発達により、節による条件、理由の表現が相対的に減少することが考えられる。

『主節』『連用節』は単回帰分析の傾きが−0.01〜0.01でcpsの変化が小さい。『主節』は「文末」「文末_その他」のみを含み、前者は動詞などの通常の述語、後者の多くは名詞で文が終わる体言止めである。これらのラベルは通常文末にのみ付与されるため、大半の文書では『主節』のcpsは 1 である。log （cps）の傾きが0に近いにも関わらず傾きが有意なのはこのためと考えられる。ただし、稀に文中に『主節』が付与されることもあるためcpsが1を超える場合がある。また、文末に「文末_理由節カラ」などが付与される場合は『理由節』などに分類されるため、cpsが1未満になる場合がある。『連用節』は『主節』と同様に log （cps）の傾きは0に近いが、『主節』よりも値のばらつきが大きく、傾きは有意ではない。『連用節』は条件節、理由節、時間節など他の連用的な節に含まれない全ての連用的な節を含むが、最も多いのは『テ』と『動詞型』 （動詞連用形節） であり、この 2 つで 67%を占める。この2つの節はいずれも等位構造を作るためによく用いられるが、cbl2におけるlog （cps）の単回帰分析 （ 表 5） では、前者が減少傾向、後者が増加傾向を示している。『動詞型』は、等位構造を作る節の中で増加傾向を示す数少ない例外である。

log （cps）の単回帰分析の結果は、全体としては学年の上昇によって等位構造が減少し、従位構造が増加することを示している。この結果は、児童はまず等位構造を使用するが次に従属節を使うようになること、述語の名詞化や修飾語化によって文を句や単語に縮約する方略を用いることになること、原因や時間を表す接続詞が減少することなどを指摘する英語での研究 （ Schleppegrell, 2004）と一致しており、日本語でも同様の傾向があると考えることができる。係り受け距離の分析結果も、この結論を支持する。等位構造的な『時間節』『条件節』『理由節』『並列節』は平均ddが3.35〜4.38と大きく、従位構造的な『引用節』『連体節』『間接疑問節』『補足節』は1.13〜2.12と小さい （ 表 6）。ddの頻度分布を単回帰分析した結果は、学年が上がるほど傾きが小さな負の値を取る （絶対値が大きくなる） ことを示している （ 表 7）。これは学年が上がるほど係り受け距離の小さい節が増えることを意味しており、従位構造が増えるというcpsの分析結果と一致する。それと同時に、学年が上がるほど係り受け距離が大きい“複雑な”文が増えるとは一概に言えないことを示唆する。

### 4.2 話し言葉から書き言葉へ

等位構造から従位構造への文構成方略の変化は『時間節』『条件節』『理由節』『並列節』などの頻度を減少させる。この変化はcbl1レベルに留まらず、それに属するcbl2レベルの節境界ラベルにも波及する。cbl2におけるlog （cps）の単回帰分析の結果 （ 表 5） は、『条件節』『理由節』『並列節』に属する主要なcbl2の多くが、cbl1と同様に減少することを示している。加えて『連用節』に属する『テ』も学年の上昇に従って減少し、唯一『動詞型』だけが増加傾向を示す。

一方で、cbl2レベルの節選択では別の変化が起こっている。cbl2の相対頻度の分析 （ 図 4） は、機能的に類似する節の間で相対的に頻度を増大させるものと減少させるものがあることを示している。最も大きな変化は、話し言葉的な節形態の減少と、それに代わる書き言葉的な節形態の増加である。特に『連用節』『条件節』『並列節』では、『テ』『タラ』『ケド』が減少し、『動詞型』『バ』『ガ』が増加している。これらの形式の文体的特性については書き言葉や話し言葉のコーパスに基づくいくつかの先行研究がある。中俣 （2015, 146） は「日本語話し言葉コーパス」と「毎日新聞」のデータで接続助詞の頻度を調査し、前者では「て」が多く、後者では「連用形」が多いことを報告している。また宮内 （2012）はBCCWJのジャンル別の接続助詞の頻度を調査し、「たら」「けれど」 （「けど」を含む） はフォーマルでない話し言葉的な形式、「ば」「が」はどのジャンルでも出現比率が高いニュートラルな形式としている。これらの研究と照らしても、作文コーパスに見られる変化はおおむね話し言葉的な節形態から書き言葉的な接形態への推移と解釈することができる。

『理由節』はもう少し複雑で、小学 4 年まで『カラ』から『ノデ』への推移が観察されたあと、中学校になると『カラ』がややシェアを取り戻す。宮内 （2012）は「から」と「ので」をいずれもフォーマルでない話し言葉的な形式としているが、一方で永野 （1952）を引用して「から」は主観的、「ので」は客観的かつ丁寧な形式としている。中学年までの『カラ』から『ノデ』への推移は、主観的な形式から客観的な形式への推移を示している可能性がある。中学校で『カラ』がシェアを取り戻す理由はまだ十分に分析できていない。「~だからだ」のような『カラ』の文末用法の増加が多少は関係しているようだが、『カラ』の再増加の全てを説明するほどには頻度が高くはない （cbl3が『文末』のものを除外しても結果は大きくは変わらない）。別の要因としては『タメ』など他の書き言葉的な理由形式が『ノデ』のシェアを奪っていることが考えられる。『タメ』はおそらく理由以外に目的を表す用法もあるため、BCCWJ-CBLでは『理由節』に分類されていないが、『カラ』『ノデ』に『タメ』を加えて学年別の相対頻度を調べると、『タメ』が小学校高学年以降シェアを増大させることが確認できる。『並列節』の『ケレド』も小学 5 年までは増加するが、その後は減少に転じる。これは話し言葉的な『ケド』からやや書き言葉的な『ケレド』への推移と、ケド・ケレド類から『ガ』への推移という 2 段階の変化が生じていることを示唆する。

cbl2の相対頻度の変化からは、少なくとも 2 つのことを読み取ることができる。第 1 に、等位構造から従位構造へという文法的方略の推移と並行して、類似する機能を持つ節のうちどの形態のものを選択するかという語彙的方略の推移が生じている。どちらの変化も、基本的には話し言葉的な文体から書き言葉的な文体への移行という大きな変化の一部であるように見える。第 2 に、節の使用傾向の変化は単に頻度のみで評価すべきではなく、機能的に類似する節の中での比率で評価する必要がある。例えば『動詞型』はcpsの分析でも増加傾向が見られるが、『テ』と比べるとシェアの増加はより顕著である。逆接の並列節では『ガ』対ケド類、『ケド』対『ケレド』という 2 つの対立があり、『ケレド』は 図 1 では増加してから減少しているが、『ケド』と『ケレド』の比率だけで見ればほぼ単調に増加している。こうした節の選択嗜好の変化は、単に個々の節の頻度 （あるいはcps） で評価するのではなく、機能的に類似する節の中での相対頻度で評価する必要がある。

加えて、これらの節選択は単に形態の選択の問題ではなく、機能の選択の問題を含んでいる可能性がある。例えば、条件節の諸形式は単に交替可能な形態的バリエーションというわけではなく、それぞれ機能の違いがある。「わからなかったら」は「わからなければ」と交替可能であり、「手をふったら」は「手をふると」と交替可能だが、「六年生になったらもっと頑張ろう」は「なれば」や「なると」と交替することはできない。従って「たら」から「ば」への移行は、単に話し言葉から書き言葉への語彙選択の変化を示しているのではなく、機能の選択の変化を包摂している可能性がある。これらの節の選択傾向をより詳細に分析するためには、どのような機能の節をよく使うかという側面と、類似する機能を持つ節のうちどの形態をよく使うかという側面を考慮しなければならない。

## データ可用性

Open Science Framework: 「児童・生徒作文コーパス」に対する節境界ラベル付与

DOI: https://doi.org/10.17605/OSF.IO/5KCRB （今田, 2020）.

このプロジェクトは以下の基本データとスクリプトを含む。

• SVMモデル作成 / 節境界ラベル付与
  - autoexec.rb （一連の処理を実行するバッチスクリプト）
  - cbl2ot.py （BCCWJ-CBLのラベル位置を修正）
  - bccwj2yamcha.py （BCCWJ形態論情報とBCCWJ-CBLを重ね合わせてYamCha形式のファイルにする）
  - yamcha_fix.py （YamCha出力結果の文字化けを修正）
  - cabocha2yamcha.rb （作文コーパスをYamCha形式のファイルにする）
  - bunsetsu.rb （統計用基本データbunsetsu.txtを作成）
• 統計処理
  - bunsetsu.txt （「児童・生徒作文コーパス」に対して節境界ラベルを付与したタブ区切りテキストファイル、本文および形態論情報を含まない）
  - metadata_1.6.txt （「児童・生徒作文コーパスVer.1.6」の統計情報を含むタブ区切りテキストファイル）
  - model_test.txt （BCCWJ-CBLによるSVMモデル評価データ）
  - statistics.Rmd / html （統計処理と図表の作成に使用したR markdownスクリプト）

以上のデータとスクリプトはクリエイティブ・コモンズ・ゼロの“No rights reserved”著作権放棄条項 （ CC0 1.0 Public domain dedication）に則って入手することができる。

SVMモデル作成と節境界ラベル付与の一連の処理を実行するには、以下の基本データが必要である。いずれも第三者のライセンスを受けたものであり、入手に制限がある。

• SVMモデル作成
  - CORE_NT/core_SUW.txt および CORE_OT/core_SUW.txt （国立国語研究所が有償で提供する「現代日本語書き言葉均衡コーパスVer.1.1 DVD版」に収録されている）
  - BCCWJ-CBL_1.0.tsv （BCCWJの利用契約者に限り、 https://chunagon.ninjal.ac.jp/ から入手できる）
• 節境界ラベル付与
  - 「児童・生徒作文コーパスVer.1.6」CaboCha 形式データ （JSPS科研費JP26285196で構築されたテキストコーパスに形態論情報を付与したもの。本文データの利用がプロジェクト関係者に限り認められているため、形態論情報の利用もそれに準ずる。）

一連のスクリプトの実行は以下の環境で行った。

• YamCha 0.33
• Python 3.8.2
• Ruby 2.7.0p0
• R 4.0.3

## Footnotes

^1^
宮城・今田 （2015）は学年が上がるにつれて1文あたりの形態素数が増加するとしているが、数値の増減は大きくなく、中学校段階では減少も見られる。

^2^
形態論情報は現在も整備を継続している。本研究で使用した版 （Ver.1.6）では、形態論情報の3分の2程度、係り受け情報の24分の1程度が手作業で修正されている。

## References

[ref1] 加納 隼人 佐藤 理史: 日本語節境界検出プログラムRainbowの作成と評価. *情報科学技術フォーラム講演論文集*. 2014;13(2):215–216.

[ref2] 金 明哲: 文節の係り受け距離の統計分析. *社会情報*. 1996;5(2):1–11. http://hdl.handle.net/10742/754

[ref3] Kudo T Matsumoto Y: Fast Methods for Kernel-Based Text Analysis. *Proceedings of the 41 ^st^ Annual Meeting on Association for Computational Linguistics.* 2003;24–31. 10.3115/1075096.1075100

[ref4] 国立国語研究所: 小学生の言語能力の発達 （国立国語研究所報告26）.明治図書. 1964. 10.15084/00001237

[ref5] Maekawa K Yamazaki M Ogiso T: Balanced Corpus of Contemporary Written Japanese. *Lang Res Eval.* 2014;48:345–371. 10.1007/s10579-013-9261-0

[ref6] 松崎 史周: 国語教育における「だらだら文」の捉え方と扱い. *日本女子体育大学紀要*. 2016;46,111–121. 10.34349/00000949

[ref7] Maruyama H Ogino S: A Statistical Property of Japanese Phrase-to-Phrase Modifications. *Mathematical Linguistics*. 1993;18(7):348–352.

[ref8] 丸山 岳彦 柏岡 秀紀 熊野 正: 日本語節境界検出プログラムCBAPの開発と評価. *自然言語処理*. 2004;11(3):39–68. 10.5715/jnlp.11.3_39

[ref9] 丸山 岳彦: BCCWJに対する節境界ラベルのアノテーション. *言語処理学会第19回年次大会発表論文集.* 2013;154–157.

[ref10] 丸山 岳彦: 現代日本語の多重的な節連鎖構造について: CSJとBCCWJを用いた分析.In: 石黒 圭 橋本 行洋[編]: *話し言葉と書き言葉の接点*. ひつじ書房.2014;93–114.

[ref11] 丸山 岳彦 佐藤 理史 夏目 和子: 現代日本語における節の分類体系について. *言語処理学会第 22 回年次大会発表論文集*. 2016;1113–1116.

[ref12] 宮城 信 今田 水穂: 『児童・生徒作文コーパス』の設計. *第7回コーパス日本語学ワークショップ予稿集*. 2015;223–232.

[ref13] 宮内 佐夜香: 接続助詞とジャンル別文体特徴の関連について: 『現代日本語書き言葉均衡コーパスを資料として』. *国立国語研究所論集*. 2012; (3),39–52. 10.15084/00000489

[ref14] 永野 賢: 「から」と「ので」とはどう違うか. *国語と国文学*. 1952;29(2):31–41.

[ref15] 中俣 尚己: 日本語並列表現の体系. ひつじ書房.2015.

[ref16] 日本語記述文法研究会[編]: 現代日本語文法6 複文. くろしお出版.2008.

[ref17] 小熊 利江 品川 直美 山下 直子: 連体修飾の使用状況に関する一考察. *言語文化と日本語教育*. 1998;16,70–79. https://teapot.lib.ocha.ac.jp/records/38946

[ref18] Schleppegrell M: *The Language of Schooling: A Functional Linguistics Perspective.* 2004; Mahwah, NJ: Lawrence Erlbaum Associates. （石川彰他[訳] : 学校教育の言語: 機能言語学の視点.ひつじ書房. 2017.）

[ref19] 張 玉潔 尾関 和彦: 文節間係り受け距離の統計的性質を用いた日本語文の係り受け解析. *自然言語処理*. 1996;4(2),3–19. 10.5715/jnlp.4.2_3

