# Supplementary material for: Annotating clause boundary labels to the written composition corpus of Japanese elementary and junior high school students
Source: F1000Res. 2021 Jul 14;10:562. [Article in Japanese] [Version 1] doi: 10.12688/f1000research.40669.1 (PMC8424459; doi:10.12688/f1000research.40669.1)
Supplement: Peer review history

# Peer review: Reviewer response for version 1

**DOI:** 10.5256/f1000research.43734.r90465

**Published:** 2021-08-19

**Authors:** Yasuhiro Kondo

**Affiliations:** 1 Aoyama Gakuin University, Tokyo, Japan

本論文は、作文コーパスに機械学習によって節ラベルを付与することを試みている。また、その結果を用いて、児童の学年進行によって、どのように節の種類に変化がみられるかを調査している。以上のように２つの課題からなっているので、査読も２つの部分に分けて示す。

 まず、機械学習（今回はSVM）によって節ラベルを付与する方法については、特に大きな問題はない。このような方法については、言語学的日本語研究においては例が少ないものであるので貴重な成果である。ただし、SVMによる節ラベルの付与の精度のヴァリデーションは、もとのBCCWJについてのテストデータで行っただけである。作文コーパスにおいて、どの程度の精度でそれが実現したかについて、何らかの言及があるとよいと思う（判定の必須条件ではなく、今後の課題あるいは小修正の可能性を示唆する）。

 次に、節の種類と学年進行の関係であるが、おおよそ事前の予想通りの結果が得られており、4節にあるような「等位構造から従位構造」への移行などかなり顕著な事象も観測され、有益である。「話し言葉から書き言葉」への推移とみる考え方にも妥当性がある。今後もこの手法によって、児童作文の分析を行うことのメリットが示されたと考える。

 以上の2点の完成度から、本論文は、適切なものとして公認されるのにふさわしい論文であるとして、承認できる。

 なお、本論文の、SVMモデル作成と節境界ラベル付与の一連の処理を実行するためには、入手に制限のあるデータが必要である。しかし、それらのデータは、第三者のライセンスにより入手できる。

（該当する場合は要回答）統計分析および解釈は適切ですか。

はい

本研究は明確かつ正確に提示されたものであり、最新の文献を引用していますか。

はい

方法と分析について第三者による再現が可能となるよう十分な詳細が提示されていますか。

はい

結果の基礎となるソースデータはすべて入手可能で再現性を十全に保証していますか。

一部該当

結論は結果により妥当な裏付けを得ていますか。

はい

研究設計は適切で学術的価値がありますか。

はい

Reviewer Expertise:

日本語文法・コーパス言語学

I confirm that I have read this submission and believe that I have an appropriate level of expertise to confirm that it is of an acceptable scientific standard.

# Peer review: Reviewer response for version 1

**DOI:** 10.5256/f1000research.43734.r89670

**Published:** 2021-08-02

**Authors:** Naoki Nakamata

**Affiliations:** 1 Kyoto University of Education, Kyoto, Japan

この論文は、著者が独自に作成した作文コーパスに節境界を自動付与し、児童・生徒の発達に伴って節の使用がどのように変化するかを探ったものです。自動付与の手法は丁寧に説明されており、今後同様の処理を行う際の参考になると思われます。分析結果については、マクロ的な節数の変化ももちろん、機能を同じくする節どうしの間の選好も発達によって変化することを明らかにしたことも、国語教育や言語学において重要な知見であり、価値が認められます。

 以下、ややわかりにくい点、疑問に感じた点を記しますので、適宜参考にしてください。

 ①「１．緒言」において、調査項目として、「節ラベル間の相対頻度」が挙げられていますが、わかりにくいです。文に対して、複数の節ラベルを付与するという文脈なので、節Aと節Bの間かと誤解してしまいました。また、２節以降では「節境界ラベルの総体頻度」というように用語が変わっています。用語をもう少し工夫し、統一なさってください。

 ②「１．緒言」に「平均的な係り受け距離は、並列節が多いほど大きく、連体節が多いほど小さくなると予想される。」とありますが、連体節が多いと小さくなるというのは機械での係り受け解析になれていない方にはなじみが薄く、少し補足説明があってもよいかと思いました。また、この問題は④の指摘とも関係があります。

 ③表 2 節境界ラベルのグループ化において、cbl１、cbl２については下位分類としてよくわかったのですが、cbl3 （接頭辞）、cbl4 （接尾辞）とは何を意味しているのかがわかりませんでした。接頭辞が「文末」というのは理解に苦しみます。作業上、このラベルが必要だったのだろうと思いますが、論文の議論としては、cbl１とcbl2のみで十分に事足りるように感じました。であれば、これらの分類を前面に出す必要はないかもしれません。

 ④「４．１等位構造から従位構造へ」の最後の段落。「これは学年が上がるほど係り受け距離の小さい節が増えることを意味しており、従位構造が増えるというcpsの分析結果と一致する。それと同時に、学年が上がるほど係り受け距離が大きい“複雑な”文が増えるとは一概に言えないことを示唆する。」

 この部分ですが、係り受け距離の定義にもよるが、「係り受け距離が長い」ことのみが複雑度が高いとは言えないのではないでしょうか。今回の分析結果は、学年が上がるほど、距離が小さくなっていますが、それは『引用節』『連体節』『間接疑問節』『補足節』が増えたからだと考えられます。確かにこれらの説は「係り受け距離」は小さいかもしれません。しかし、それは日本語が構造的に埋め込まれた節の主要部が、埋め込み先の名詞の直前に来るということで、表面的な現象であると思います。実際に『引用節』『連体節』『間接疑問節』『補足節』を多用した文は明らかに複雑です。埋め込みの深さなど、認知的負荷は増しています。「係り受け距離が増えるとは言えない」とは言えると思いますが、それをもって「複雑な文が増えるとは一概に言えない」とは言えないのではないでしょうか。

 ⑤「ノデ」節の変化は非常に興味深いです。「主観から客観」という議論が行われていますが、これは文体とも関係があるのではないでしょうか。「ので」はより丁寧な形式とされており、丁寧体の文章と相性が良いと思われます。よって、一旦は「ので」が増加するが、作文の文体が発達につれて「です・ます体」ではなくなっていくのにしたがって、「ので」の使用も減少するのでは、と考えました。

（該当する場合は要回答）統計分析および解釈は適切ですか。

はい

本研究は明確かつ正確に提示されたものであり、最新の文献を引用していますか。

はい

方法と分析について第三者による再現が可能となるよう十分な詳細が提示されていますか。

はい

結果の基礎となるソースデータはすべて入手可能で再現性を十全に保証していますか。

はい

結論は結果により妥当な裏付けを得ていますか。

はい

研究設計は適切で学術的価値がありますか。

はい

Reviewer Expertise:

日本語学、計量言語学

I confirm that I have read this submission and believe that I have an appropriate level of expertise to confirm that it is of an acceptable scientific standard.
